# Switchable Vanadium Dioxide Metasurface for Terahertz Ultra-Broadband Absorption and Reflective Polarization Conversion

**DOI:** 10.3390/mi15080967

**Published:** 2024-07-28

**Authors:** Wei Zou, Changqing Zhong, Lujun Hong, Jiangtao Lei, Yun Shen, Xiaohua Deng, Jing Chen, Tianjing Guo

**Affiliations:** 1 Department of Physics, School of Physics and Material Science, Nanchang University, Nanchang 330031, China; 2 Institute of Space Science and Technology, Nanchang University, Nanchang 330031, China; 3 School of Physics, Nankai University, Tianjin 300071, China; 4 Collaborative Innovation Center of Extreme Optics, Shanxi University, Taiyuan 030006, China

**Keywords:** VO_2_, terahertz metasurface, ultra-broadband absorption, reflective polarization conversion

## Abstract

Based on the unique insulator-metal phase transition property of vanadium dioxide (VO_2_), we propose an integrated metasurface with a switchable mechanism between ultra-broadband absorption and polarization conversion, operating in the terahertz (THz) frequency range. The designed metasurface device is constructed using a stacked structure composed of VO_2_ quadruple rings, a dielectric layer, copper stripes, VO_2_ film, a dielectric layer, and a copper reflection layer. Our numerical simulations demonstrate that our proposed design, at high temperatures (above 358 K), exhibits an ultra-broadband absorption ranging from 4.95 to 18.39 THz, maintaining an absorptivity greater than 90%, and achieves a relative absorption bandwidth of up to 115%, significantly exceeding previous research records. At room temperature (298 K), leveraging VO_2_’s insulating state, our proposed structure transitions into an effective polarization converter, without any alteration to its geometry. It enables efficient conversion between orthogonal linear polarizations across 3.51 to 10.26 THz, with cross-polarized reflection exceeding 90% and a polarization conversion ratio over 97%. More importantly, its relative bandwidth reaches up to 98%. These features highlight its wide-angle, extensive bandwidth, and high-efficiency advantages for both switching functionalities. Such an ultra-broadband convertible design offers potential applications in optical switching, temperature dependent optical sensors, and other tunable THz devices in various fields.

## 1. Introduction

Recently, metasurfaces [1,2,3,4,5,6] have gained widespread interest for their ability to freely manipulate the amplitude, phase, and polarization of electromagnetic waves at the sub-wavelength scale, because of the appealing advantages including compactness, flexibility, broad bandwidth, and easy integration [7,8,9,10]. At THz wavelengths, metasurfaces can exhibit a range of distinctive properties that are useful in developing various functional devices, such as beam splitters, biosensors, filters, polarization converters, optical switches [11,12,13,14], and absorbers [15,16,17,18,19]. Conventional metasurfaces are constructed from metallic or dielectric materials with dimensions much smaller than the wavelength of operation, which allows the subwavelength structures to alter the polarization, phase, amplitude, and frequency of electromagnetic waves. However, the characteristics of traditional metasurfaces are generally fixed post-manufacture. To create flexible THz metasurfaces, active materials such as graphene [20,21,22], VO_2_ [23,24,25], Ge_2_Sb_2_Te_5_(GST) [26,27], and photoconductive semiconductors [28,29] have been integrated into traditional metasurfaces, significantly enhancing their tunability. Notably, graphene stands out owing to its dynamically adjustable carrier mobility and conductivity [30,31,32]. A switchable broadband absorber and reflector was developed using a hybrid graphene-gold metasurface, achieving high absorption from 0.53 to 1.05 THz over wide incidence angles [33]. VO_2_ is renowned for its unique metal-insulator phase transition characteristics and has extraordinary appeal in THz applications, which has led to its application in the development of thermally tunable metasurfaces, marking significant progress in this field [34,35,36]. Moreover, among the aforementioned phase-change materials, VO_2_ stands out as a distinctive material, featuring multiple modulation modes such as electrical, thermal, or optical excitation, with an extremely short response time (approximately 100 fs) and a remarkable modulation depth. This is mainly attributed to the fact that when the temperature climbs to approximately 358 K, its structural phase transforms from the monoclinic phase to the tetragonal phase, causing a substantial change in the dielectric constant. Incorporating VO_2_ into THz metasurfaces allows for the creation of adjustable multifunctional devices, significantly impacting the development of THz technology. For example, the concurrent absorption and polarization conversion functionalities can enhance THz imaging and sensing techniques, while the wavefront manipulation can facilitate beam steering and shaping in THz communication and imaging systems [37,38].

A wide operation bandwidth is always desired for effective performance in practical applications. Broadband absorption, for instance, is crucial for solar energy capture and light detection, achievable through the layering of structures with differing absorption characteristics or the amalgamation of materials with various absorption mechanisms. Reports have emerged of a broadband switchable THz half-/quarter-wave plate based on metal-VO_2_ metamaterials, demonstrating dual functionality within 0.66 to 1.44 THz range [39]; furthermore, a transition from a broadband perfect absorber to a broadband polarization converter has been achieved, with bifunctionality spanning 0.52 THz to 1.04 THz [40]; additionally, another actively switchable VO_2_ metasurface has been engineered for concurrent absorption and polarization conversion switching, effectively manipulating the functionalities from 1.41 to 2.69 THz [41]. With the increasing demands in integrated and multifunctional devices, broadband switching phenomena are highly desirable and promising, but still challenging in the THz range.

In this study, an ultra-broadband versatile THz metasurface device is proposed by designing a stacked structure. By utilizing the phase-change capabilities of VO_2_, the device can switch between broad-spectrum absorption and polarization conversion. For the absorption mode corresponding to VO_2_ in the metallic state, the device achieves an ultra-broadband absorption across 4.95 to 18.39 THz, with absorption greater than 90%. For the polarization conversion mode corresponding to VO_2_ in the insulating state, it acts as a polarization converter within 3.51 to 10.26 THz, with more than 90% efficiency in cross-polarized reflection. Additionally, we carried out numerical simulations to investigate the effects of the polarization, incident angle, and the device geometry on the versatile device. This structure outshines previous switchable metasurfaces by offering ultra-broadband capabilities, wide-angle functionality, efficient conversion, and polarization-sensitive absorption, holding great promise in optical communications, THz polarization sensors, optical switching, and beyond.

## 2. Structure Design and Numerical Method

**Figure 1:**
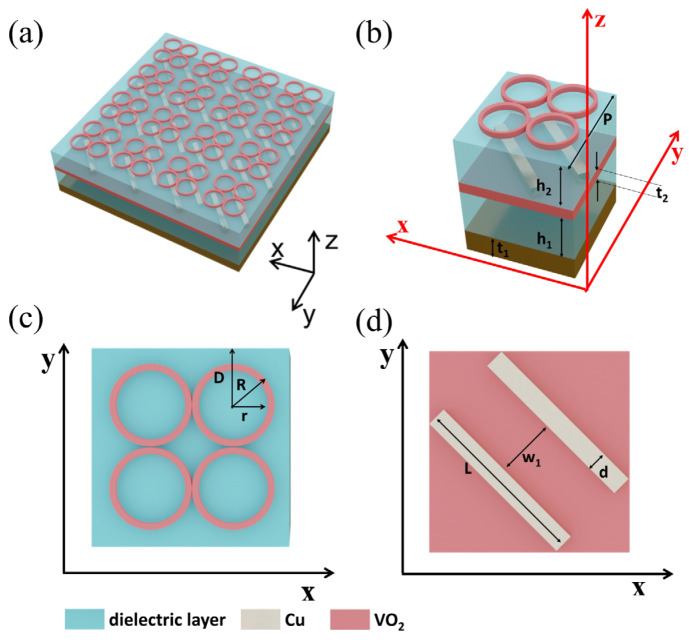
(**a**) Schematic structure of the proposed VO_2_ metasurface structure with switchable functions of ultra-broadband absorption and polarization conversion in THz range. (**b**) The 3D schematic of one unit cell of the stacked structure, consisting of six layers from top to bottom: VO_2_ quadruple ring, dielectric layer, Cu stripes, VO_2_ film, dielectric layer, and Cu reflection layer. The optimized geometry parameters are P = 20 μm, h_1_ = 7.4 μm, h_2_ = 5 μm, t_1_ = 0.2 μm, and t_2_ = 0.1 μm. (**c**) The top view of a unit cell. The parameters of the VO_2_ quadruple ring are optimized with outer and inner radii R = 4 μm, and r = 3 μm, respectively. The distance from the ring center to the unit cell border is D = 6 μm. (**d**) The parameters of the middle Cu stripes. Their parameters are optimized as L = 18 μm and d = 2 μm. The spacing between the two strips is w_1_ = 5.35 μm.

We consider the quadruple ring in our metasurface design to realize a polarization-independent THz absorber with VO_2_ in insulating state. Periodic oblique copper (Cu) stripes are placed in the middle of the stacked structure to play an important role in the polarization conversion when VO_2_ stays in a fully metallic state at room temperature. The 3D structure for the proposed design and its unit cell are schematically depicted in Figure 1a and Figure 1b, respectively, which consists of six layers from top to bottom: the VO_2_ quadruple ring, a dielectric layer, Cu stripes, VO_2_ film, a dielectric layer, and a Cu reflection layer. The corresponding geometric parameters are presented in the caption of Figure 1b–d. To effectively optimize the proposed structure design, we employ the finite-difference time-domain (FDTD) method to explore its electromagnetic response. Since the proposed design is periodic in the x and y directions, we simulate just one unit cell numerically to speed up both the operation and analysis. Periodic boundary conditions are used along the x and y directions, with open boundary conditions along the z direction to generate incident wave on the structure. Through rigorous optimization, the period along the x and y directions is fixed at P = 20 μm, with the dielectric layer thickness being h_1_ = 7.4 μm, h_2_ = 5 μm. Additionally, the thicknesses of the Cu reflection layer and the VO_2_ film are t_1_ = 0.2 μm and t_2_ = 0.1 μm, respectively. Figure 1c illustrates the parameter of the VO_2_ quadruple ring, with outer and inner radii R = 4 μm and r = 3 μm, respectively. The distance from the center to the border is D = 6 μm. From Figure 1d, it can be seen that the Cu stripes are positioned at an angle of 45° relative to the x-axis, each having a length of L = 18 μm, a width of d = 2 μm, a spacing between the two strips of w_1_ = 5.35 μm, and a thickness of t_4_ = 0.1 μm. These parameters are fixed unless stated otherwise.

In the THz frequency range, the relative permittivity of VO_2_ can be described with the Drude model [42,43,44,45]:

(1) $$\varepsilon_{{\mathrm{VO}}_{2}}(\omega )=\varepsilon_{\infty }-\frac{\omega_{p}^{2}}{\omega (\omega +i\gamma )}$$

where $\omega_{p}$ is the plasma frequency, calculated by ${\omega_{p}}^{2}={\omega_{p0}}^{2}\cdot \sigma /\sigma_{0}$. The initial value of the plasma frequency is $\omega_{p0}=1.45\times 10^{15}\;\mathrm{rad}/s$, $\sigma_{0}=3\times 10^{5}\;S/m$. Here, $\varepsilon_{\infty }=12$ is the dielectric permittivity in the infinite frequency, and the collision frequency $\gamma =5.75\times 10^{13}\;\mathrm{rad}/s$. Given that the conductivity σ is proportional to the free space carrier density, it fluctuates with the phase change of the VO_2_ film. In our numerical simulations, we adjust the conductivity values to reflect the VO_2_ material’s varying states. In this study, it is important to note that we only examine the fully metallic and fully insulating states of VO_2_ in order to achieve switchable functionalities of the proposed structure. Specifically, when the VO_2_ is in its metallic state, it possesses a conductivity of 2 × 10^5^ S/m, and when in the insulator state, its conductivity is 10 S/m. The dielectric layer consists of a depleted polymer with a relative dielectric constant of ε = 1.5 + 0.005i. The material Cu is set as a lossy metal with an electrical conductivity of 5.8 × 10^7^ S/m.

## 3. Results and Discussion

### 3.1. Ultra-Broadband Absorption with the VO_2_ Metasurface Structure

**Figure 2:**
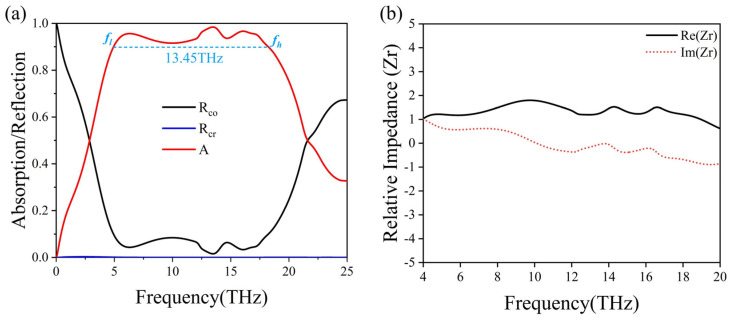
(**a**) Simulated absorption, cross- and co-polarized reflection spectra of the proposed VO_2_ metasurface structure under y-polarization incidence, when VO_2_ is fully in a metallic state. (**b**) Real (black line) and imaginary (red dashed line) parts of the calculated relative effective impedance of the proposed VO_2_ metasurface structure.

In order to verify the performance of the proposed structure, we investigate the configuration shown in Figure 1 by employing numerical simulations. The electric field of the incident electromagnetic wave is polarized along the y-axis. We first calculate the absorption spectra when VO_2_ quadruple ring and VO_2_ film are in a completely metallic state. The absorption of the proposed structure can be obtained by A = 1 − R − T, where R and T are the computed reflection and transmission, respectively. The continuous VO_2_ film remains in its metallic state, acting as a reflective ground plane, and effectively prevents any transmission. As a result, the absorption can be computed with A = 1 − R. Here, the cross- and co-polarized reflection, i.e., R_cr_ and R_co_, are also demonstrated for comparison with the results from the polarization conversion function (see details in the next subsection). The simulated spectra are presented in Figure 2a, as a function of frequency. It can be clearly observed that ≥90% absorption is achieved in a broad frequency range from 4.95 to 18.4 THz. The relative absorption bandwidth (RAB) is defined to enable an effective comparison of bandwidth characteristics, expressed as $\mathrm{RAB}=2\times \left(f_{h}-f_{l}\right)/\left(f_{h}+f_{l}\right)$, where *f_h_* and *f_l_* are the upper and lower frequency bounds, respectively, corresponding to frequencies at which the absorption exceeds 90%. Understanding the definition of RAB is crucial for making accurate bandwidth comparisons across different operational frequencies. By calculation, it is determined that the structure’s RAB can achieve up to 115.2% when VO_2_ is fully in a metallic state, indicating that this metasurface design functions as an effective absorber over a wide frequency range in the THz spectrum.

To thoroughly comprehend the absorption mechanism of the proposed structure, we utilize the impedance matching theory [46,47]. According to the theory, the absorption of the structure can be calculated by using $A=1-|(Z-Z_{0})/(Z+Z_{0}){|}^{2}$, where Z_0_ is the free space impedance, and Z is the effective impedance of the proposed structure, described as $Z=\sqrt{\mu_{1}/\varepsilon_{1}}$, where $\mu_{1}$ and $\varepsilon_{1}$ stand for the effective permeability and permittivity, respectively. The absorption formula can be simplified to $A=1-|(Z_{r}-1)/(Z_{r}+1){|}^{2}$ by utilizing the relative impedance $Z_{r}=Z/Z_{0}$, which can be computed with S-parameters from our numerical simulations, $Z_{r}=\sqrt{\left({\left(1+S_{11}\right)}^{2}-{S_{21}}^{2})/({\left(1-S_{11}\right)}^{2}-{S_{21}}^{2}\right)}$. In a two-port network, S_11_ represents the reflection at port 1 when the incident wave enters port 1, and S_21_ represents the transmission from port 1 to port 2. As a result, S_11_ and S_22_ are the reflection and transmission coefficients of the incident electromagnetic wave. The simplified formulae indicate that complete absorption can be realized when the relative impedance is near 1, meaning that the structure’s impedance should match to the free space impedance. The broadband absorption is confirmed in Figure 2b through the calculation of the real and imaginary parts of the relative impedance of the structure. It is evident that the real and imaginary parts of the absorber’s relative impedance are approximately 1 and 0, respectively, across a wide frequency range. This clearly demonstrates the successful achievement of impedance matching between the proposed structure and the free space.

**Figure 3:**
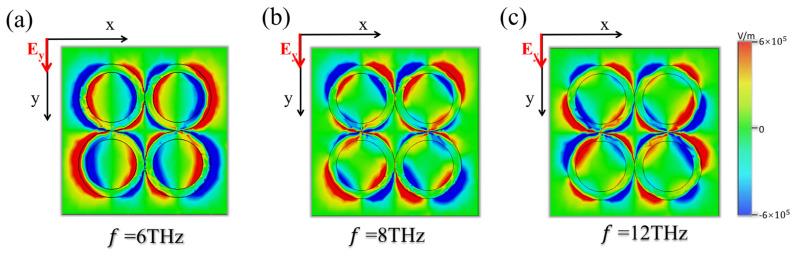
The computed electric field distributions of the ultra-broadband absorber with y-polarized incidence at (**a**) 6 THz, (**b**) 8 THz, and (**c**) 12 THz with the VO_2_ in the metallic state.

**Figure 4:**
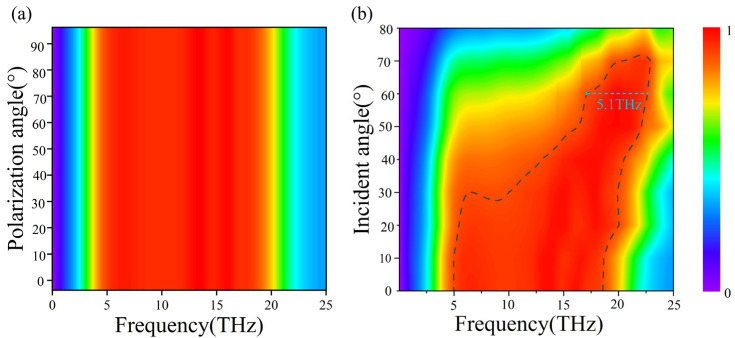
The computed absorption contour plots of the proposed VO_2_ metasurface structure as a function of the frequency and (**a**) polarization angles; (**b**) incident angles of the excitation wave under y-polarization.

We further illustrate the fundamental mechanism of broadband absorption by calculating the electric field intensity distribution of the structure. Figure 3 depicts the field distribution with y-polarization incidence on the structure. We have selected three frequencies (6 THz, 8 THz, and 12 THz) to aid in analyzing the broadband mechanism of the absorption spectrum. Upon observing the corresponding E_z_ components at these resonances, we find that the incident energy is localized along the VO_2_ ring in all the cases. At the resonance frequency f_1_ = 6 THz, the length of one ring in the structure is approximately equal to half the wavelength of the electromagnetic field, and electric dipole resonance manifests in each ring, as shown in Figure 3a, which explains the high absorption at this frequency. At frequencies f_2_ = 8 THz and f_3_ = 12 THz, the length of one ring in the structure is approximately equal to two half-waves of the electromagnetic field, and quadrupole resonance occurs in each ring, contributing to a high absorption, as depicted in Figure 3b,c, respectively. It is important to mention that in the proposed stacked structure, the broadband absorption is not affected by polarization of incident waves, thanks to the symmetry of the structure. The simulated results are presented in Figure 4a, where absorption contour plots are calculated as a function of frequency and polarization angles. The absorption remains its peak value between 4.95 and 18.4 THz regardless of the polarization.

Next, we investigate the performance of the proposed absorber for different incident angles of the excitation wave. The ability to deal with wide incident angles is a critical criterion for assessing absorbers. The calculated absorption is shown in Figure 4b as a function of the incident angle and operation frequencies. It is evident that the absorption bandwidth remains wide when the incident angle is below 50°, and the absorption can reach a high value of 93.2% with the incident angle being 70°. However, the absorption bandwidth gradually decreases when the incident angle is beyond the 50° threshold. Importantly, the ≥90% absorption bandwidth can reach up to 5.1 THz even though the incident angle is as high as 60°. These findings illustrate that the proposed design is capable of attaining high-efficiency absorption along with broad-angle and extensive bandwidth features.

**Figure 5:**
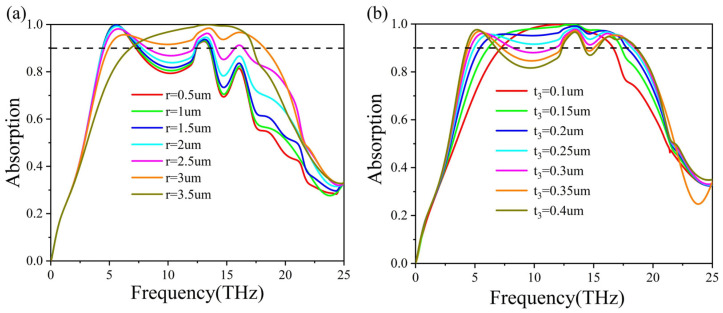
The simulated absorption of the proposed VO_2_ metasurface structure by varying (**a**) inner radius r, (**b**) thickness t3 of the VO_2_ ring. The VO_2_ material is in completely metallic state with a conductivity value of σ = 2 × 10^5^ S/m.

We further consider how the broadband absorption spectrum varies with the structure’s geometrical features. Based on the initial analysis (structure design and field distribution), we have determined that the quadruple VO_2_ ring plays a crucial role in achieving complete absorption. As a result, our focus is on the thickness and the inner radius of the VO_2_ ring positioned atop the metasurface structure. Figure 5a illustrates the effect of the inner radius r on the absorption spectra. It is found that as the radius r increases from 0.5 μm to 3.5 μm, the ≥90% absorption gradually transitions from multiple peaks to a single peak, with the first peak diminishing gradually. The maximum bandwidth (≥90%) is achieved at r = 3 μm, culminating in a perfect absorption with a single peak at 3.5 μm. In Figure 5b, we calculate the absorption spectra with different thickness t_3_ of the VO_2_ ring. It can be seen that as t3 increases from 0.1 μm to 0.4 μm, the maximum absorption decreases from 100% to 90%. Simultaneously, the absorption bandwidth increases until t_3_ reaches 0.25 μm. At around 10 THz, the absorption of the structure starts to decline as the thickness of the VO_2_ ring increases, resulting in the loss of the broad absorption characteristics. To conclude, the parameters of the VO_2_ ring are important for the achievement of its broadband absorption. Therefore, careful consideration of the structure’s geometry is essential for future experimental verification.

### 3.2. Effective Polarization Converter with the Proposed Metasurface Structure

**Figure 6:**
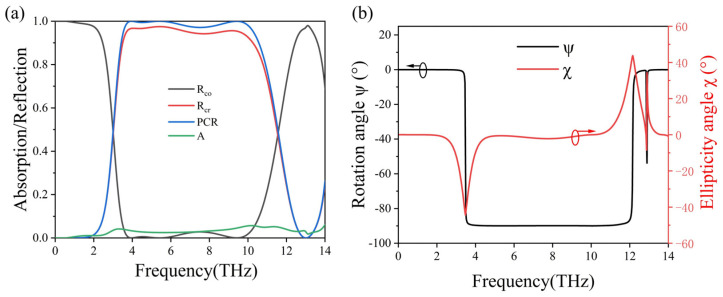
(**a**) The simulated co-polarized (R_co_), cross-polarized (R_cr_) reflection, PCR and the absorption (A) of the proposed VO_2_ metasurface structure under y-polarized plane wave excitation, when VO_2_ is in fully insulating state with a conductivity value of s = 10 S/m. (**b**) The computed polarization rotation angle y and ellipticity c of the proposed structure under normal incidence.

By manipulating the external temperature of the metasurface, the phase transition of VO_2_ from the metallic state to the dielectric state is accomplished. Once a fully insulating state is attained, VO_2_ enables our structure to shift from functioning as an absorber to operating as a polarizer, without the requirement of any geometrical alterations. In this case, the conductivity of the VO_2_ material is σ = 10 S/m. To investigate the structure’s polarization conversion efficiency, we calculate the cross-polarized (R_cr_) and co-polarized (R_co_) reflection as a function of frequency. Since the electric field of the incident electromagnetic wave is polarized along the y-axis, the cross-polarization R_cr_ [48] is defined as $R_{\mathrm{cr}}=\left|E_{x}^{\mathrm{Reflec}}/E_{y}^{\mathrm{Inc}}\right|$, where $E_{y}^{\mathrm{Inc}}$ is the electric field of the y-polarized incident waves and $E_{x}^{\mathrm{Reflec}}$ is the x component of the reflected waves. Similarly, the co-polarization is expressed as $R_{\mathrm{co}}=\left|E_{y}^{\mathrm{Reflec}}/E_{y}^{\mathrm{Inc}}\right|$, where $E_{y}^{\mathrm{Reflec}}$ is the y component of the reflected waves. We define the polarization conversion ratio (PCR) as PCR = R_cr_/(R_co_ + R_cr_) to further reveal the efficiency of the proposed polarization converter. As Figure 6a clearly indicates, the cross-polarization R_cr_ consistently outperforms the co-polarization R_co_ over a wide frequency spectrum, ranging from 3.51 to 10.26 THz. Notably, the PCR remains above 97% across this extensive frequency range, as highlighted by the blue line in Figure 6a. As a result, the relative bandwidth is up to 98%. We also present the absorption spectrum of the structure in Figure 6a for comparison with the findings depicted in Figure 2a. This juxtaposition between Figure 6a and Figure 2a clearly demonstrates the structure’s transition from a broadband absorber to a wideband polarizer. 

To accurately characterize the polarization state of the reflective polarization converter, the Stokes method is introduced [49,50], and four parameters are defined as:

(2) $$\begin{array}{l} S_{0}=r_{xy}^{2}+r_{yy}^{2}\\ S_{1}=r_{xy}^{2}-r_{yy}^{2}\\ S_{2}=2r_{xy}r_{yy}cos\left(\Delta \varphi \right)\\ S_{3}=2r_{xy}r_{yy}sin\left(\Delta \varphi \right) \end{array}$$

where *r_ij_* (*i*, *j* = *x*, *y*) is the calculated i-polarized reflection coefficient from the j-polarized incidence. Δφ is the phase difference of *r_xy_* and *r_yy_*. The benchmark for pure linear polarization conversion is determined by the polarization rotation angle ψ and the ellipticity angle χ, which can be computed by Stokes parameters in Equation (2):

(3) $$\begin{array}{l} tan2\psi =S_{2}/S_{1}\\ \chi =arcsin\left(S_{3}/S_{0}\right) \end{array}$$

Figure 6b illustrates the polarization rotation angle and ellipticity angle when a y-polarized plane wave is excited under normal incidence. The polarization rotation angle ψ, represented by the black line, remains at −90° across a wide frequency range from 3.5 to 12 THz. This signifies that the reflected wave undergoes approximately a 90° rotation after passing through the proposed structure, transitioning from a y-polarized incident wave. Additionally, the ellipticity angle χ maintains near 0° between 4.8 to 10.26 THz, indicating that the reflected wave maintains a very high purity of linear polarization. These findings suggest that the designed structure effectively converts a y-polarized incident wave to a nearly pure x-polarized wave over a broad frequency range.

**Figure 7:**
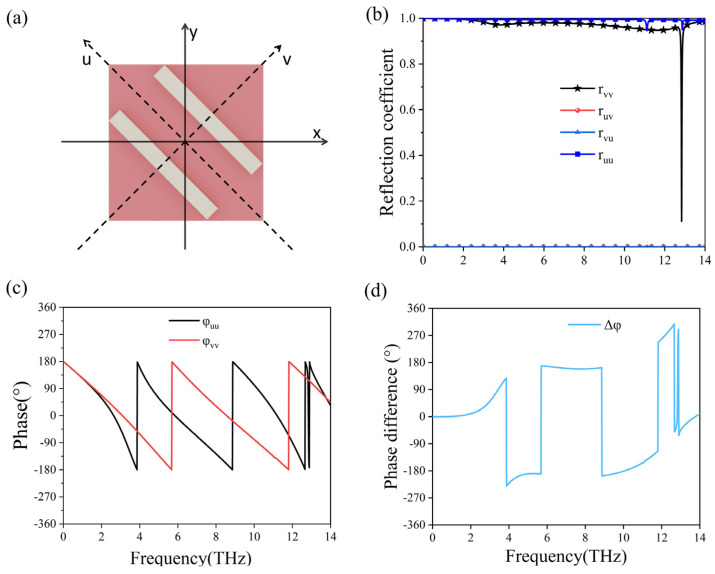
(**a**) Schematic diagram of the orthogonal decomposition of the metasurface polarizer into two components along the u- and v-axes. (**b**) The simulated reflection coefficients of the decomposed reflected wave, r_vv_, r_uv_, r_vu_, and r_uu_. (**c**) The computed phase of the reflection coefficients r_uu_ and r_vv_. (**d**) Phase difference between the reflection coefficients r_uu_ and r_vv_.

We conduct an investigation of the polarization conversion mechanism by examining the formation of a Fabry–Perot cavity with the Cu substrate and an array of Cu strips [44], since the current VO_2_ material is in a fully insulting state. The subsequent interference from polarization coupling during multiple reflections can be attributed to the enhancement or reduction of the total reflected field involving co-polarization and cross-polarization. For easier analysis, we orthogonally split the incident y-polarized wave into two components along the u-axis and the v-axis, as shown in Figure 7a. We calculate the respective reflections rij (where i, j = u, v), indicating the reflection of an i-polarized wave from a j-polarized incident wave. The findings, illustrated in Figure 7b, reveal that both r_uu_ and r_vv_ values remain above 0.9 across the entire computation range, except for a notable dip in reflection at around 13 THz. Conversely, the reflections r_uv_ and r_vu_ are so minimal that they are practically negligible. Consequently, our analysis focuses solely on the phases $\varphi_{uu}$, $\varphi_{vv}$, and their phase difference Δφ, as displayed in Figure 7c,d. Figure 7d highlights that the phase difference spans from 4.2 to 11.78 THz, roughly equating to 180° or −180°. This suggests that the combined reflected waves are diverted by 90° relative to the incoming waves.

**Figure 8:**
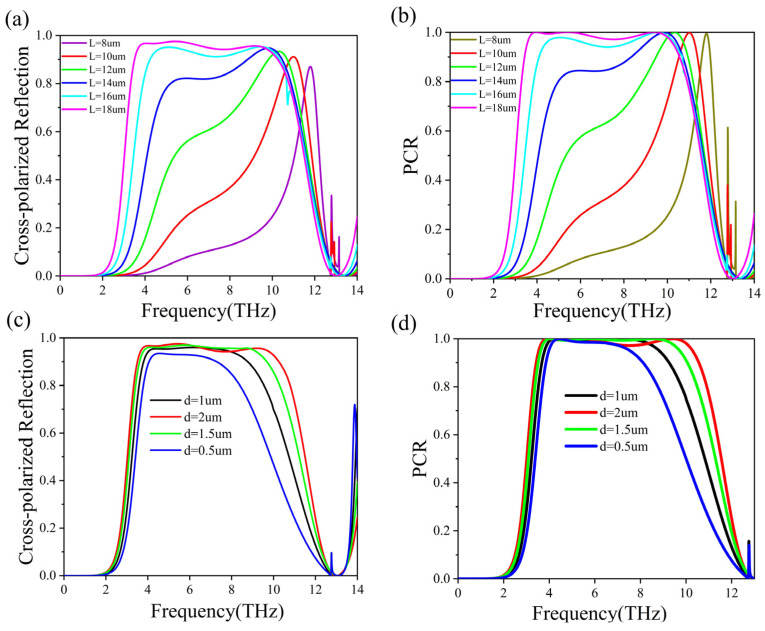
(**a**,**b**) The simulated (**a**) cross-polarized reflection, (**b**) PCR of the proposed structure by varying the length L of the Cu strips. (**c**,**d**) The simulated (**c**) cross-polarized reflection, (**d**) PCR of the proposed structure by varying the width d of the Cu strips. VO_2_ is in fully insulating state with a conductivity value of σ = 10 S/m.

As we have analyzed, the linear polarization converter is largely attributed to the design of the Cu strips and the Cu reflective layer on the bottom. We delve into how the Cu strips—specifically, the length L and the width d—affect the reflection of cross-polarized light and the PCR. As illustrated in Figure 8a, we observe that as the Cu strip length L increases from 8 μm to 18 μm, the bandwidth for cross-polarization progressively broadens. Concurrently, the entire spectral profile exhibits a red shift, with the lower frequency end elevating until it aligns with the higher frequency end. This results in an expansion of the cross-polarization bandwidth, which attains its maximum when L equals 18 μm. Figure 8b illustrates the evolution of the PCR as the length L of the Cu strips increases from 8 μm to 18 μm. During this process, the PCR spectral profile evolves from a singular peak to a broadband configuration characterized by multiple peaks, mirroring the cross-polarization observed in Figure 8a. Throughout this transformation, the entire spectral line experiences a red shift, while the elevation in the lower frequency region gradually nears unity, ultimately resulting in the emergence of a broadband spectral line. Similarly, the impact of the Cu strip width d on the cross-polarization reflection amplitude is explored in Figure 8c. As the width d increases from 0.5 μm to 2 μm, the amplitude of the cross-polarization reflection steadily increases, stabilizes, and its bandwidth broadens, ultimately achieving maximum bandwidth. As shown in Figure 8d, the amplitude of the PCR remains essentially constant, while the bandwidth progressively widens. From this parameter analysis, it is concluded that both the length and width substantially impact the polarization converter’s bandwidth, with the variation in length L exerting a more pronounced effect on the conversion efficiency.

**Figure 9:**
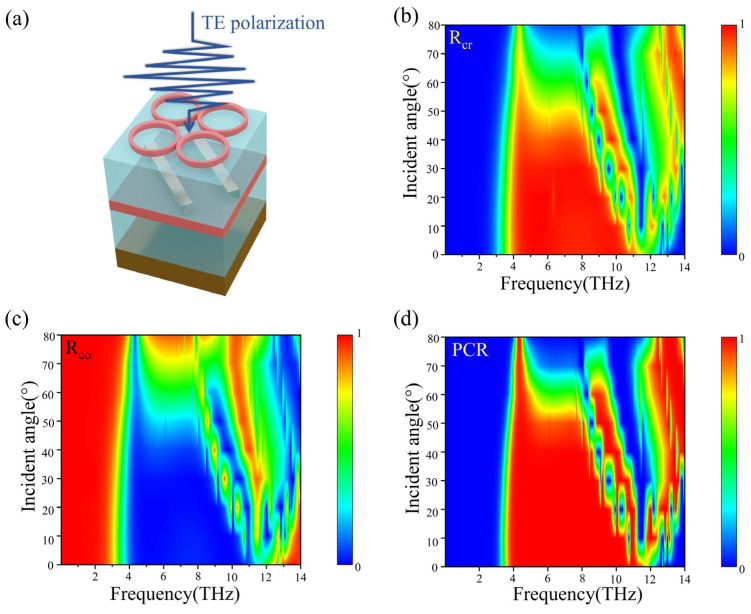
(**a**) Schematic diagram of one unit cell under y-polarization incidence. The computed contour plots of the (**b**) cross-polarized reflection, (**c**) co-polarized reflection, and (**d**) PCR as a function of the frequency and incident angles of the excitation wave in y-polarization when VO_2_ is in a fully insulating state with a conductivity value of σ= 10 S/m.

Finally, to gain a deeper insight into the characteristics of the linearly polarized converter, we simulate the reflection coefficient and PCR at different incidence angles under y-polarization, as depicted in Figure 9a. Figure 9b shows the color map of the cross-polarized reflection R_cr_ as a function of the incidence angle and frequency, demonstrating that the reflection bandwidth of the cross-polarized reflection (>90%) incrementally narrows as the incidence angle is increased from 0 to 50°. The magnitude of the cross-polarized reflection decreases when the angle of incidence exceeds 50°. In Figure 9c, we observe that the co-polarized reflection R_co_ climbs as the angle of incidence increases within the operational frequency range, yet remains under 0.1 for angles of incidence below 50°. In addition, we observe that the impact on the PCR value is minimal with an incident angle smaller than 50°, and the bandwidth begins to narrow progressively. From these observations, it can be deduced that the designed metasurface structure is capable of delivering robust linear polarization performance at incidence angles up to 50°. This suggests that the linear polarization converter can sustain its linear polarization functionality effectively even at larger angles of incidence.

As has been documented, there is extensive research on multifunctional devices utilizing active materials. This study highlights the broad bandwidth achieved for both absorption and polarization conversion using the same structure. To evaluate our metasurface structure’s bandwidth, we compared its absorption and polarization conversion bandwidth, along with their relative bandwidths, to those of other notable multifunctional devices reported recently. Table 1 shows that our devices outperform others in terms of broader absorption and polarization conversion capabilities, along with a larger relative bandwidth. Moreover, our device presents the benefits of being more compact and featuring a thinner thickness, indicating a wider scope of potential applications.

## 4. Conclusions

In summary, we have demonstrated a versatile THz metasurface that can switch from ultra-broadband absorption to linear polarization conversion by use of the metal-to-insulator transition of VO_2_. Results have shown that when VO_2_ is in the metallic state, the metasurface device acts as an ultra-broadband absorber, covering a spectrum from 4.95 to 18.39 THz, and is unaffected by polarization, working well even at large incident angles. By changing the phase of VO_2_ into the insulating state, the device becomes a polarization converter with broadband cross-polarization reflection across 3.51 to 10.26 THz. This enables a seamless switch between absorption and polarization conversion over a wide frequency range from 4.95 to 10.26 THz, by electrically, thermally, or optically adjusting the VO_2_ state, without altering the device’s geometry. The findings presented in this paper demonstrate the advantages of the proposed stacked structure, including switchable functions, ultra-broadband operation, and efficient conversion, suggesting its potential applications in optical switching, temperature-sensitive sensors, and other tunable THz devices in various fields.

## Figures and Tables

**Table 1:** Comparison with recently reported multifunctional metasurfaces.

Ref.	Principal Material	Absorption Bandwidth [THz]	Relative Bandwidth	Polarization Conversion Bandwidth PCR > 90% [THz]	Relative Bandwidth	Thickness [μm]
[51]	VO_2_	(1.03–2.62) 1.59	87.1%	(1.81–2.39) 0.58	27.6%	42.5
[52]	VO_2_	(1.80–4.05) 2.25	76.9%	(1.68–3.79) 2.11	77.1%	25.9
[53]	VO_2_	(1.97–4.63) 2.66	80.6%	(1.54–4.18) 2.64	92.3%	23.45
[54]	VO_2_, Graphene	0.74; 1.45	-	(0.335–1.275) 0.94	116.77%	85.5
[55]	VO_2_	(0.562–1.232) 0.67	74.7%	(0.63–1.12) 0.49	56%	76.3
[56]	VO_2_, PS *	(0.68–1.6) 0.92	80.70%	(0.82–1.6) 0.78	64.46%	48.6
This work	VO_2_	(4.95–18.39) 13.44	115.1%	(3.51–10.26) 6.75	98%	12.95

* PS represents photoconductive semiconductor.

## Data Availability

Data underlying the results presented in this paper are not publicly available at this time but may be obtained from the authors upon reasonable request.
